# COP I vesicles facilitate classical swine fever virus proliferation by transporting fatty acid synthase from the Golgi apparatus to the endoplasmic reticulum

**DOI:** 10.1128/jvi.00305-25

**Published:** 2025-06-03

**Authors:** Liang Zhang, Tao Wang, Chen Chen, Mengzhao Song, Ning Li, Bihao Luo, Yuehan Quan, Kangkang Guo, Yanming Zhang

**Affiliations:** 1 College of Veterinary Medicine, Northwest A&F University 718173, Yangling, Shaanxi, China; 2 State Key Laboratory for Animal Disease Control and Prevention, College of Veterinary Medicine, Lanzhou University, Lanzhou Veterinary Research Institute, Chinese Academy of Agricultural Sciences 111658 , Lanzhou, Gansu, China; Loyola University Chicago - Health Sciences Campus, Maywood, Illinois, USA

**Keywords:** classical swine fever virus, early secretory pathway, coatomer protein I, coatomer protein II, fatty acid synthase

## Abstract

Classical swine fever virus (CSFV) is an enveloped, positive-sense, single-stranded RNA virus in the *Flaviviridae* family that remodels the cell’s endomembrane for its own propagation. The early secretory pathway is exploited by viruses for their lifecycle, but the mechanism underlying this hijacking of the early secretory pathway in CSFV infection remains unknown. Here, we observed that disrupting the functions of the early secretory pathway organelles, the Golgi apparatus, the endoplasmic reticulum (ER), and coatomer protein I (COP I) vesicles and coatomer protein II (COP II) vesicles resulted in a significant inhibition of CSFV propagation. Further, we revealed that COP I vesicles were required for CSFV RNA replication, but not for the formation of viral replication complexes. The results support the hypothesis that participation of COP I vesicles in viral RNA replication involves their capacity for cargo trafficking. Intact COP I vesicles were isolated and subjected to data-independent acquisition quantitative proteomics analysis to identify the differences in the proteomes of COP I vesicles. This analysis revealed an increase in fatty acid synthase (FASN), a critical factor for CSFV RNA replication, within COP I vesicles, while its presence in COP II vesicles decreased in CSFV-infected cells. Meanwhile, blocking COP I vesicle formation resulted in decreased levels of FASN in the ER, impairing CSFV RNA replication. Collectively, we provide evidence that COP I vesicles mediate FASN trafficking from the Golgi apparatus to the ER to facilitate CSFV RNA replication, which advances our understanding of the role of the early secretory pathway in CSFV proliferation.

**IMPORTANCE:**

Classical swine fever is a highly contagious disease caused by the classical swine fever virus (CSFV) that infects domestic pigs and wild boars and results in significant economic losses to the swine industry. The early secretory pathway in host cells has often been hijacked by viruses for viral genome replication, assembly, and release of virions. Here, our data revealed that the function of early secretory pathway organelles such as the endoplasmic reticulum (ER) and the Golgi apparatus, and the membrane-bound transport intermediates, COP I vesicles and COP II vesicles, that facilitate transport, were involved in CSFV proliferation in PK-15 cells. Our findings demonstrate that COP I vesicles significantly promote CSFV RNA replication by trafficking fatty acid synthase from the Golgi apparatus to the ER. Our data suggest that manipulation of early secretory pathway function in target host cells could provide a promising strategy for a novel anti-CSFV therapeutic.

## INTRODUCTION

Classical swine fever (CSF), caused by the classical swine fever virus (CSFV), is a highly infectious disease that necessitates notification of the World Organization for Animal Health due to its significant impact on the swine industry (1). CSFV is a member of the *Flaviviridae* family and possesses a single-stranded, positive-sense RNA genome of about 12.3 kb. During CSFV infection, the viral RNA genome is directly translated into a large polyprotein consisting of 3,898 amino acids, which is subsequently cleaved by viral and host proteases to yield four structural proteins (C, E^rns^, E1, and E2) and eight nonstructural proteins (N^pro^, p7, NS2, NS3, NS4A, NS4B, NS5A, and NS5B) (2).

The early secretory pathway is a highly conserved and complex network that mediates the transport of newly synthesized proteins from the endoplasmic reticulum (ER) to the Golgi apparatus through the endoplasmic reticulum Golgi intermediate compartment (ERGIC). It consists of the structurally distinct organelles, the ER, ERGIC, and Golgi apparatus, and specific membrane-bound transport intermediates known as coatomer protein I (COP I) vesicles and coatomer protein II (COP II) vesicles that facilitate intracellular transport (3). In the ER, newly synthesized proteins undergo folding and initial glycosylation through interactions with chaperones. At the ER exit sites, the proteins bind to COP II through N-terminal signal sequences and then are sequestered in COP II vesicles for transport to the ERGIC (4). After arriving at the ERGIC, COP II vesicles release their cargo, which undergoes further modification and maturation. Tubular vesicles are responsible for transporting proteins along microtubules to the Golgi apparatus. During this process, the COP I vesicles transport proteins from the Golgi apparatus back to the ER (5, 6).

The early secretory pathway is an essential cellular apparatus conserved across all eukaryotes and involved in cellular organization, dynamics, and homeostasis. Not surprisingly, it is often hijacked by viruses for accomplishing the main steps in their lifecycle, including entry (7), viral genome replication (8), and release (9). RNA viruses, such as those in the *Coronaviridae* and *Flaviviridae* families, which replicate in the cytoplasm, are often associated with the early secretory pathway, using it to get to the Golgi apparatus before leaving the cell (10). Hepatitis C virus (HCV), a member of the same *Flaviviridae* family as CSFV, forms cup-shaped replication organelles (ROs) that protrude from ER membranes (11). The Golgi apparatus is involved in the formation of HCV ROs as Golgi-specific brefeldin A-resistance factor 1 (GBF1) is involved in HCV replication (12). Li and colleagues reported that COP I vesicles facilitated HCV RNA replication by mediating phosphatidylinositol-4-phosphate (PI4P) accumulation in HCV ROs (13). After envelopment, HCV is trafficked from the ER to the Golgi apparatus in COP II vesicles similar to those used by cellular cargos (9). Severe acute respiratory syndrome coronavirus 2 (SARS-CoV2), which belongs to the *Coronaviridae* family, forms double-membrane vesicles (DMVs), which are generated from the ER and act as platforms for viral RNA replication (14). The virions are assembled at the ER or the ERGIC and are thought to use lysosomes for release, but not the early secretory pathway (15, 16). The COP II vesicles promote egress of the newly made spike protein from the ER into the secretory pathway by binding to it, and COP I vesicles carry spike proteins through retrograde trafficking back to the progeny assembly site in the ERGIC (17, 18). These reports suggested that the early secretory pathway supported viral proliferation in different ways.

Our previous studies demonstrated that the early secretory pathway regulatory proteins, GBF1, ADP-ribosylation factor 1 (ARF1), ADP ribosylation factor GTPase-activating protein 1 (AFRGAP1), Ras-related protein 1 (Rab1), and Ras-related protein 2 (Rab2), were required for CSFV infection (19–22). However, the roles of all the components of the early secretory pathway in the proliferation of CSFV are still not fully understood. Here, systematic experiments were conducted to decipher the role of the main components of the early secretory pathway in the lifecycle of CSFV, and we found that CSFV infection induced morphological alterations of early secretory pathway organelles, disrupting the function of COP I and COP II vesicles and inhibiting CSFV infection. Mechanistically, inhibition of the formation of COP I vesicles impaired CSFV RNA replication by inhibiting fatty acid synthase (FASN), an essential factor for CSFV RNA replication, transport from the Golgi apparatus to the ER. Our findings provide valuable insights into the role of COP I vesicles in CSFV RNA replication, which advances our understanding of the role of the early secretory pathway in regulating CSFV infection.

## RESULTS

### Disrupting early secretory pathway organelles inhibits CSFV proliferation

**Fig 1:**
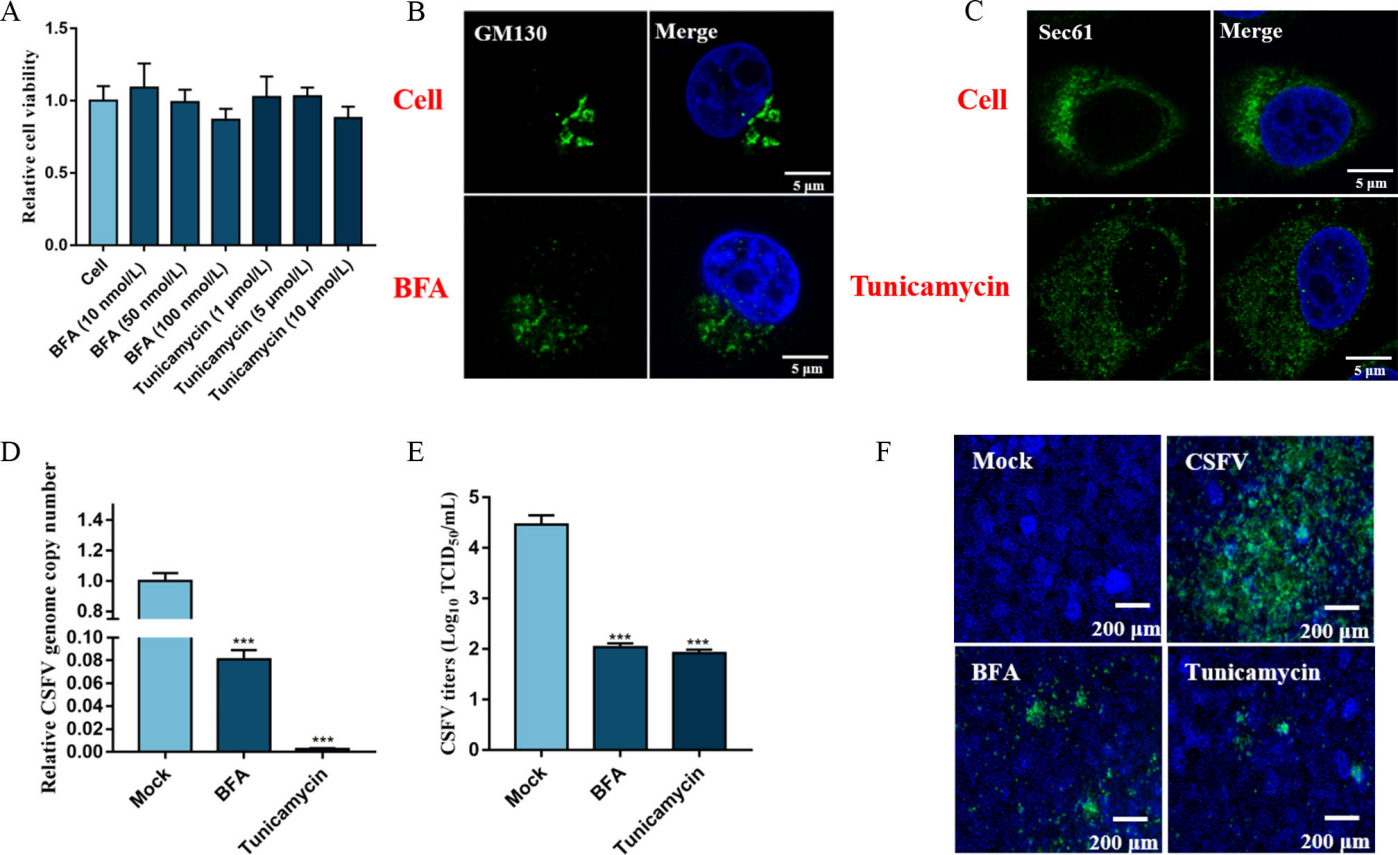
Disrupting the functions of early secretory pathway organelles inhibits CSFV infection. (**A**) CCK-8 assays were conducted to measure the viability of cells after treatment with BFA and tunicamycin. (**B**) PK-15 cells were treated with or without 100 nM BFA for 24 h, cells were fixed in 4% paraformaldehyde, and immunofluorescence staining was performed using an anti-GM130 antibody. Scale bars: 5 µm. (**C**) PK-15 cells were treated with or without 10 µM tunicamycin for 24 h, cells were fixed in 4% paraformaldehyde, and immunofluorescence staining was performed using an anti-Sec61 antibody. Scale bars: 5 µm. (D and E) PK-15 cells were co-treated with 1 MOI CSFV and 10 µM tunicamycin or 100 nM BFA for 48 h, and then cell and culture supernatants were collected for determination of CSFV RNA copy numbers and virus titers by RT-qPCR and TCID_50_/mL, respectively. (**F**) PK-15 cells were co-treated with 1 MOI CSFV and 10 µM tunicamycin or 100 nM BFA for 48 h, and then cells were fixed in 4% paraformaldehyde and stained with anti-E2 antibody (green). Scale bars, 200 µm.

To evaluate the influence of early secretory pathway organelles on CSFV infection, we tested two cytotoxic compounds, tunicamycin, an ER stress inducer that inhibits protein glycosylation and disrupts normal ER function, and brefeldin A (BFA), an inhibitor of ER-to-Golgi transport that rapidly damages the structure of the Golgi apparatus. Measurements of cell viability showed that 10 µM tunicamycin and 100 nM BFA were not toxic to PK-15 cells (Fig. 1A). However, when PK-15 cells were treated with 10 µM tunicamycin or 100 nM BFA for 24 h, the ER and the Golgi apparatus were examined by confocal microscopy, they were observed to have changed from perinuclear aggregation to cytoplasmic dispersion (Fig. 1B and C), confirming that BFA and tunicamycin caused dysfunction of the Golgi apparatus and the ER. Next, the PK-15 cells were infected with 1 MOI CSFV and incubated with 100 nM BFA or 10 µM tunicamycin for 48 h. After incubation, the cells and supernatants were harvested for quantification of viral genome copy number and viral titration. As shown in Fig. 1D and E, CSFV propagation was significantly reduced in the BFA- and tunicamycin-treated cells (*P* < 0.001). Additionally, the proliferation of CSFV in the cells was determined by immunofluorescence assay (IFA). The viral plaques in BFA- and tunicamycin-treated cells were smaller than those of the control cells (Fig. 1F). Altogether, these data demonstrate that normal functioning of the Golgi apparatus and the ER is essential for CSFV infection.

### COP I and COP II vesicles positively regulate CSFV propagation

**Fig 2:**
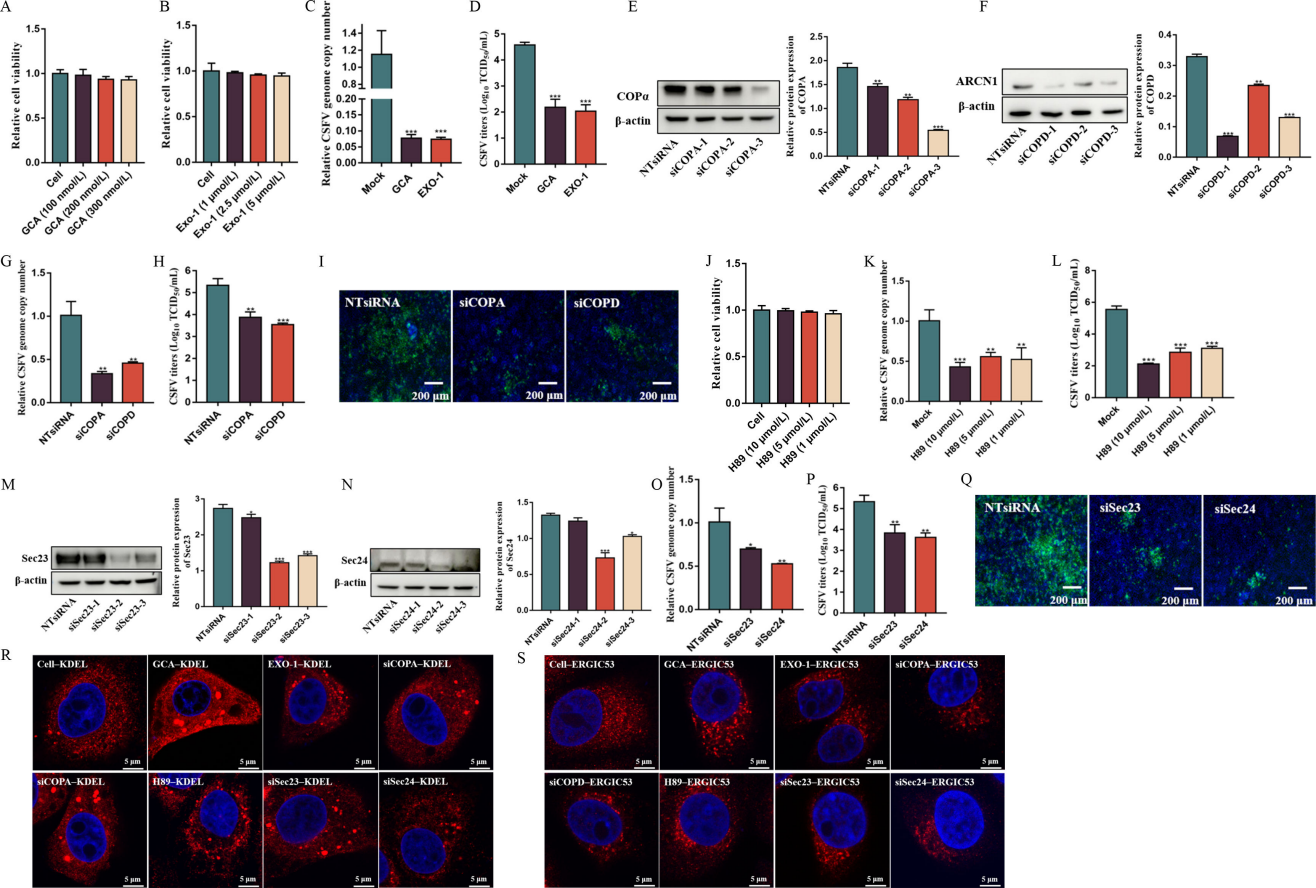
COP I and COP II positively regulate CSFV propagation. (A and B) CCK-8 assays were conducted to measure the cell viability after treatment with GCA and Exo-1. (C and D) PK-15 cells were pretreated with 300 nM GCA or 5 µM Exo-1 for 24 h and then infected with 1 MOI CSFV. After 48 h, the cells and culture supernatants were harvested for determining CSFV RNA copy numbers and virus titers by RT-qPCR and TCID_50_/mL, respectively. (E and F) Cells were transfected with NTsiRNA, siCOPA-1, siCOPA-2, siCOPA-3, siCOPD-1, siCOPD-2, and siCOPD-3 for 48 h. Cells were harvested, and COPα and ARCN1 levels were quantitated by Western blot. (G and H) Cells transfected with NTsiRNA, siCOPA-3, and siCOPD-1 were infected with 1 MOI CSFV. Then, cells and culture supernatants were harvested for measurement of CSFV RNA copy numbers and virus titers by RT-qPCR and TCID_50_/mL. (**I**) NTsiRNA-, siCOPA-3-, or siCOPD-1-transfected cells were infected with 1 MOI of CSFV. After 48 h, the cells were fixed in 4% paraformaldehyde and stained with anti-E2 antibody (green). Scale bars, 200 µm. (**J**) CCK-8 assays were conducted to measure the viability of cells after treatment with H89. (K and L) PK-15 cells were pretreated with various concentrations of H89 for 24 h and then infected with 1 MOI of CSFV. After 48 h, cell and culture supernatants were harvested for measuring CSFV RNA copy numbers and virus titers by RT-qPCR and TCID_50_/mL, respectively. (M and N) Cells were transfected with NTsiRNA, siSec23, and siSec24 for 48 h and then harvested for determination of Sec23 and Sec24 levels by Western immunoblot assay. (O and P) NTsiRNA-, siSec23-2-, and siSec24-2-transfected cells were infected with 1 MOI of CSFV, and after 48 h, the cells and culture supernatants were harvested for measurement of CSFV RNA copy numbers and virus titers by RT-qPCR and TCID_50_/mL, respectively. (**Q**) NTsiRNA-, siSec23-2-, and siSec24-2-transfected cells were infected with 1 MOI of CSFV, and after 48 h, the cells were fixed in 4% paraformaldehyde and stained with anti-E2 antibody. Scale bars, 200 µm. (**R**) CSFV (1 MOI)-infected cells were treated with GCA, Exo-1, and H89 or transfected with siCOPA, siCOPD, siSec23, and siSec24 for 48 h, and the distribution of the KDEL was viewed under confocal microscopy. (**S**) CSFV (1 MOI)-infected cells were treated with GCA, Exo-1, and H89 or transfected with siCOPA, siCOPD, siSec23, and siSec24 for 48 h, and the distribution of the ERGIC53 was viewed under confocal microscopy. Scale bars: 5 µm.

After determining that ER and Golgi apparatus function were important for CSFV infection, we examined whether COP I and II vesicles also contributed to viral production. COP I vesicles coordinate the trafficking of cell components from the Golgi apparatus to the ER (6). Therefore, we conducted experiments to uncover the function of COP I vesicles in CSFV infection of PK-15 cells. The toxicity of two COP I inhibitors, golgicide A (GCA) and Exo-1, on PK-15 cells was first determined by viability measurements using a CCK-8 kit (Fig. 2A and B). Then, PK-15 cells were separately pretreated with 300 nM GCA and 5 µM Exo-1 for 24 h and infected with 1 MOI CSFV. After 48 h of CSFV infection, samples were harvested for quantification of the number of copies of the viral genome and the virus titer. RT-qPCR and IFA assays revealed that GCA and Exo-1 markedly suppressed CSFV proliferation (*P* < 0.001; Fig. 2C and D). To further determine the effects of COP I vesicles on CSFV infection, siRNA sequences against COPA and COPD were designed and transfected into PK-15 cells. After 48 h, the silencing efficacy was measured by Western blot assay, with siCOPA-3 and siCOPD-1 showing the greatest knock-down efficacy (*P* < 0.001; Fig. 2E and F). The siCOPA-3- and siCOPD-1-transfected cells were inoculated with 1 MOI CSFV, and, after 48 h, we observed that the genetic interference with COPA and COPD significantly reduced CSFV proliferation (*P* < 0.01; Fig. 2G and H). CSFV propagation in cells was also determined by IFA, and the CSFV plaques in siCOPA-3- and siCOPD-1-transfected cells were smaller and fewer than those in controls (Fig. 2I). These findings demonstrate that impairing COP I vesicle formation inhibited CSFV propagation.

COP II vesicles, which traffic proteins from the ER to the Golgi apparatus, are composed of Sec23-Sec24 heterodimers and Sec13-Sec31 heterotetramers (23). To investigate the participation of COP II vesicles in CSFV proliferation, PK-15 cells were pretreated with a range of concentrations of H89 (10, 5, and 1 µM), a specific inhibitor of COP II, for 24 h, and then infected with 1 MOI CSFV. After 48 h, the cells and supernatants were harvested for quantification of viral genome copy number and virus titer. We saw a dose-dependent inhibition of CSFV propagation by H89 (*P* < 0.01; Fig. 2J, K, and L). Next, siRNA sequences against Sec23 and Sec24 were synthesized and transfected into PK-15 cells. After 48 h, the silencing efficacy was measured using Western immunoblotting, with siSec23-2 and siSec24-2 showing the greatest knockdown (*P* < 0.001; Fig. 2M and N). Next, siSec23-2- and siSec24-2-transfected cells were inoculated with 1 MOI of CSFV. After 48 h, cells and cell supernatants were harvested for CSFV proliferation determination using RT-qPCR and TCID_50_/mL. The results revealed that genetic silencing of Sec23 and Sec24 caused a significant reduction in CSFV proliferation (*P* < 0.05; Fig. 2O and P). Additionally, CSFV propagation in cells was also determined by IFA. The CSFV plaques of siSec23-2- and siSec24-2-transfected cells were smaller and fewer than those in control cells (Fig. 2Q). These results indicate that COP II vesicles significantly contribute to CSFV propagation.

We evaluated whether the role of COP I vesicles and COP II vesicles in the proliferation of CSFV depends on its mediated protein transport function between the ER and Golgi apparatus. The localization of KDEL and ERGIC53 in GCA-, Exo-1, and H89-treated cells and in siCOPA-, siCOPD-, siSec23-, and siSec24-transfected cells was viewed by confocal microscopy. We found that treatment with either inhibitors or siRNA targeting COP I vesicles or COP II vesicles disrupted KDEL and ERGIC53 distribution, suggesting the effects of COP I vesicles and COP II vesicles in the proliferation of CSFV depend on its mediated protein transport function between the ER and Golgi apparatus (Fig. 2R and S).

### COP I vesicles are required for CSFV RNA replication

**Fig 3:**
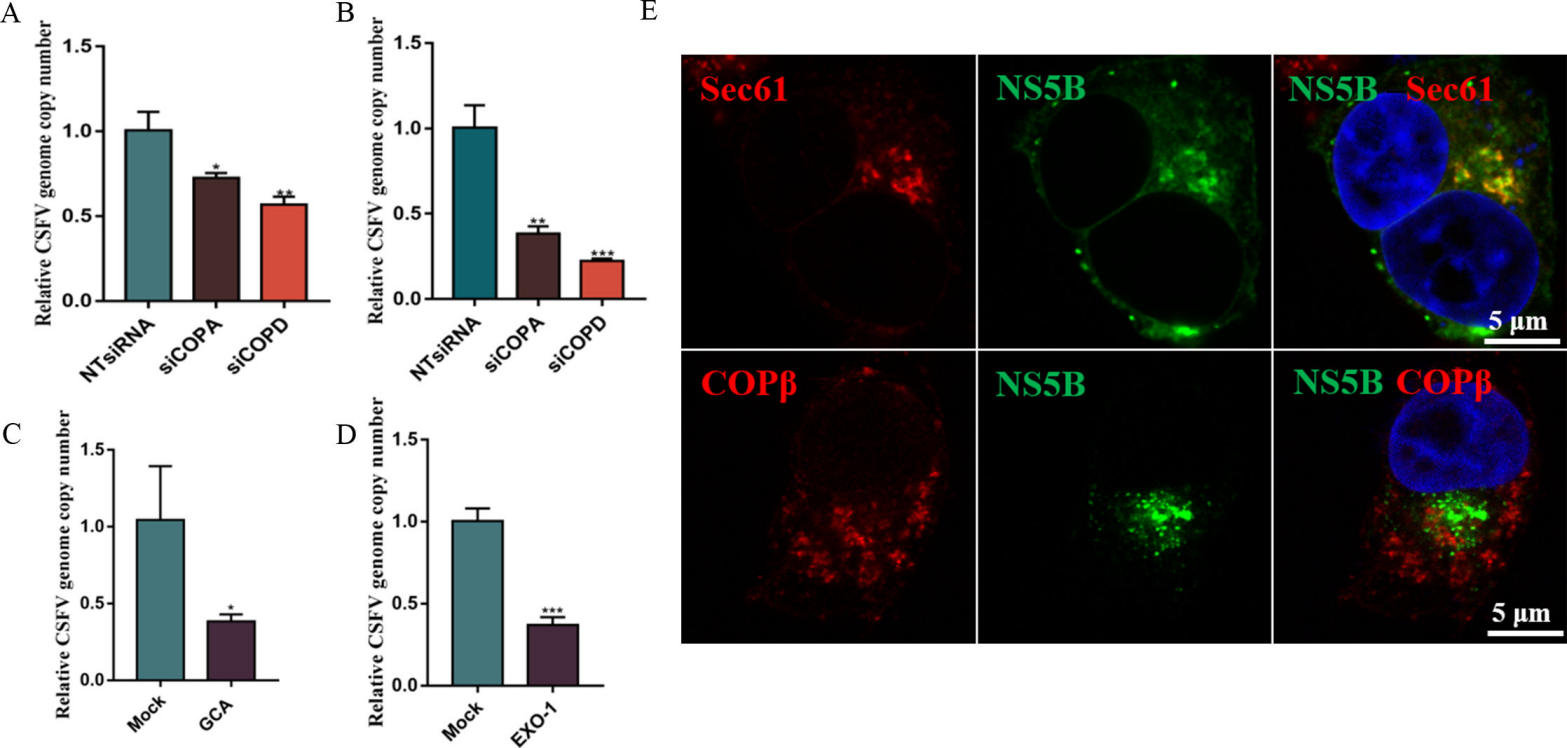
COP I is required for CSFV RNA replication. (**A**) NTsiRNA-, siCOPA-3-, and siCOPD-1-transfected cells were infected with 10 MOI of CSFV in FBS-free medium for 1 h at 4°C. Unbound virions were washed away with pre-cooled citrate buffer (pH = 3). Total cells were collected for CSFV RNA copy number measurement by RT-qPCR. (**B**) NTsiRNA-, siCOPA-3-, and siCOPD-1-transfected cells were infected with 10 MOI CSFV (MOI = 10) in FBS-free medium for 1 h at 4°C to allow virion binding. Cells were then washed with pre-cooled citrate buffer (pH = 3) to remove unbound virions and cultured for another 2 h at 37°C. The cells were washed and collected for CSFV RNA copy number determination by RT-qPCR. (C and D) PK-15 cells were infected with 1 MOI of CSFV for 2 h, then the medium was discarded, and the fresh medium containing GCA or Exo1 was added. Samples were collected after 8 h incubation for CSFV RNA copy number determination by RT-qPCR. (**E**) The pEGFP-NS5B-transfected cells were infected with 1 MOI of CSFV for 48 h. Cells were stained for the COP I vesicle marker, COPβ, the ER marker, Sec61, and nucleocapsids. Scale bars: 5 µm.

Considering the positive effects of COP I vesicles on CSFV proliferation, experiments were carried out to identify which stages of the CSFV life cycle were affected by COP I vesicles in PK-15 cells. To test viral binding, cells transfected with siCOPA-3 and siCOPD-1 were inoculated with CSFV (10 MOI) in FBS-free medium and incubated for 1 h at 4℃. Unbound virions were washed away with cold citrate buffer (pH 3). The cells were harvested, and an RT-qPCR assay revealed that the number of copies of the virus genome was lower in siCOPA-3- and siCOPD-1-transfected cells, demonstrating that COP I vesicles were necessary for CSFV binding (*P* < 0.05; Fig. 3A). For virus entry, siCOPA-3- and siCOPD-1-transfected cells were inoculated with 10 MOI of CSFV and cultured in FBS-free medium for 1 h at 4℃ to allow virion attachment. Cells were rinsed with cooled citrate buffer (pH 3) to remove unbound virions and cultured for another 2 h at 37°C. The cells were extensively washed, and RNA was isolated to determine the numbers of copies of the CSFV genome. The RT-qPCR assay revealed that CSFV genome copy numbers were lower in siCOPA-3- and siCOPD-1-transfected cells (*P* < 0.01; Fig. 3B), demonstrating that COP I vesicles were necessary for CSFV entry. These results confirm that COP I vesicles play an essential role in CSFV binding and entry.

The silencing of COPA and COPD impairs the entry and proliferation of CSFV; thus, the effect of inhibition of COP I vesicles on other CSFV lifecycle stages may be caused by the reduction in CSFV invasion. Therefore, specific inhibitors GCA and Exo-1 of COP I vesicles were used to study the role of COP I vesicles in CSFV RNA replication. To exclude the effect of inhibitors on CSFV invasion, cells were infected with CSFV for 2 h. Then, the medium was discarded, and the fresh medium containing GCA and Exo-1 was added. Cells were collected after 8 h incubation. As shown in Fig. 3C and D, we found that CSFV genome copy numbers decreased in GCA- and Exo-1-treated cells (*P* < 0.05), which suggests that blocking COP I vesicle formation inhibits viral RNA replication. As COP I vesicles have the function of providing membrane, we hypothesized that COP I vesicles may be required for the assembly of the virus replication complex. However, confocal microscopy did not show COP I vesicles colocating with CSFV NS5B (Fig. 3E), a CSFV replication complex located protein, suggesting that COP I vesicles are required for CSFV RNA replication, but not for viral replication complex formation.

### Characterization of COP I and II vesicles by proteomics

**Fig 4:**
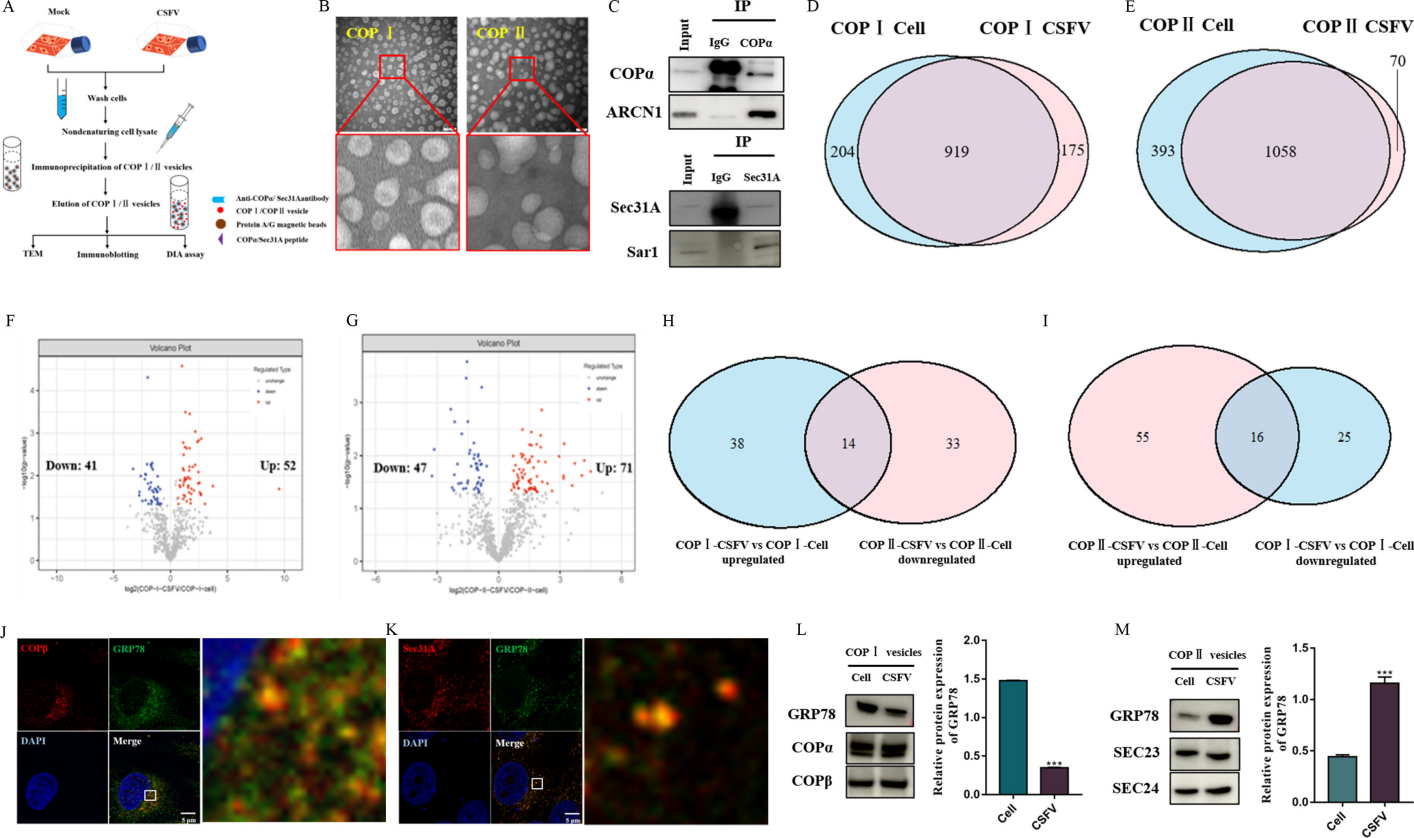
Proteomics characterization of isolated COP I and COP II vesicles. (**A**) Schematic representation of the experimental timeline for isolation and purification of COP I and COP II vesicles. (**B**) The isolated COP I and COP II vesicles were analyzed by negatively stained TEM. (**C**) The isolated COP I and COP II vesicles were analyzed by Western blot. (**D**) Venn diagram showing proteins in common identified in COP I vesicles from CSFV-infected and noninfected cells. (**E**) Venn diagram of proteins in common identified in COP II vesicles from CSFV-infected and noninfected cells. (**F**) Volcano plot showing differences in protein abundance from COP I vesicles isolated from CSFV-infected and noninfected cells. (**G**) Volcano plot showing protein abundance differences in COP II vesicles between CSFV-infected and noninfected cells. (**H**) Venn diagram of common proteins in upregulated proteins of COP I vesicles (CSFV vs noninfected) and downregulated proteins of COP II vesicles (CSFV vs noninfected) (**I**) Venn diagram of common proteins in downregulated proteins of COP I vesicles (CSFV vs noninfected) and upregulated proteins of COP II vesicles (CSFV vs noninfected). (**J**) Colocalization of GRP78 and COPβ. Cells were infected with CSFV (MOI = 1) for 48 h and stained for GRP78, COPβ, and nucleocapsid. Scale bars: 5 µm. (**K**) Colocalization of GRP78 and Sec31A. Cells were infected with CSFV (MOI = 1) for 48 h and stained for GRP78, Sec31A, and nucleocapsid. Scale bars: 5 µm. (**L**) The GRP78 levels in isolated COP I vesicles from CSFV-infected and uninfected cells were analyzed by Western blot. (**M**) The GRP78 levels in isolated COP II vesicles from CSFV-infected and uninfected cells were analyzed by Western blot.

Since COP I vesicles are not directly involved in the CSFV RNA replication, we hypothesized that the vesicles may be dependent on their protein transport function to regulate the CSFV RNA replication. To test our hypothesis, COP I and COP II vesicles were isolated from CSFV-infected cells and non-infected cells by immunoprecipitation with anti-COPα and anti-Sec31A antibodies that bind specifically to the outer coat of COP I and II vesicles, respectively (Fig. 4A). Isolated vesicles were characterized by transmission electron microscopy (TEM) and identified using Western blot. The TEM results showed many intact vesicular structures with an average diameter of 50–100 nm (Fig. 4B). Western immunoblotting showed that the isolated vesicles contained the COP I components, COPα and ARCN1, and the COP II components, Sec31A and Sar1 (Fig. 4C). These findings indicated that immunoprecipitation with the anti-COPα and anti-Sec31A antibodies effectively enriched intact COP I and COP II vesicles, respectively. Then, data-independent acquisition (DIA) quantitative proteomics was performed to identify the differences in the proteomes of COP I and COP II vesicles from CSFV-infected and noninfected cells. A total of 1,094 proteins were separately quantified in COP I vesicles from CSFV-infected and 1,123 proteins from noninfected cells (Table 1; Table S1 and S2). A total of 1,128 proteins were separately quantified in COP II vesicles from CSFV-infected cells and 1,451 proteins from noninfected cells (Table 1; Table S3 and S4). In total, 1,298 proteins were identified and quantified in COP I vesicles from CSFV-infected and noninfected cells, and 919 proteins were common to both groups (Fig. 4D; Table 1 and 2; Table S5). In COP II vesicles, 1,521 proteins were identified and quantified from CSFV-infected and noninfected cells, and 1,058 proteins were common to both groups (Fig. 4E; Table 1 and 2; Table S6). In addition, the 1,040 overlap proteins were shared in COP I vesicles and COP II vesicles in uninfected cells, and 951 overlap proteins were shared in COP I vesicles and COP II vesicles in CSFV-infected cells (Table S7 and S8).

**TABLE 1:** Total identified proteins

Sample	No. of proteins identified	Total no. of proteins identified
COPI-Cell	1,123	1,298
COPI-CSFV	1,094	
COPII-Cell	1,451	1,521
COPII-CSFV	1,128	

**TABLE 2:** Results of differentially expressed protein analysis

Sample comparison	Total no. of proteins identified	No. of upregulated proteins	No. of downregulated proteins	Total no. of DEFs
COPI-CSFV vs COPI-cell	919	52	41	93
COPII-CSFV vs COPII-cell	1,058	71	47	118

As shown in Fig. 4F and Table 2, our results revealed a shift in the quantitative proteome composition in COP I vesicles from CSFV-infected cells, reflected by 93 differentially expressed proteins (DEPs), of which 52 were upregulated and 41 were downregulated, compared with COP I vesicles from noninfected cells (*P* < 0.05; CSFV/cell > 1.5 or <0.67). Additionally, we also showed a shift in the quantitative proteome composition in COP II vesicles from infected cells, reflected in 118 DEPs, of which 71 were upregulated and 47 were downregulated, compared with COP II vesicles from noninfected cells (*P* < 0.05; CSFV/cell > 1.5 or <0.67) (Fig. 4G; Table 2). Since COP I is involved in CSFV RNA replication, and CSFV RNA replication occurs in the ER, we hypothesized that proteins located in the Golgi apparatus, which are involved in replication of the CSFV RNA, may be transported to the ER through COP I vesicles. These proteins may be reduced in COP II vesicles to accumulate in the ER, where they promote CSFV RNA replication. Therefore, common upregulated proteins in COP I vesicles (CSFV vs noninfected) and downregulated proteins of COP II vesicles (CSFV vs noninfected) were analyzed (Fig. 4H; Table 3). Common proteins among downregulated proteins from COP I vesicles (CSFV vs noninfected) and upregulated proteins of COP II vesicles (CSFV vs noninfected) are also shown in Fig. 4I and Table 4. Then, the GRP78, which is transported between the Golgi apparatus and the ER by COP I and COP II vesicles, was used to verify the accuracy of the proteomics. Confocal microscopy showed that GRP78 colocalized with COP I and COP II vesicles in CSFV-infected cells (Fig. 4J and K). The relative content of GRP78 in COP I and COP II vesicles was quantified by Western immunoblotting assay and revealed that the level of GRP78 in COP I vesicles from CSFV-infected cells was significantly lower than that from noninfected cells (Fig. 4L), while GRP78 in COP II vesicles from CSFV-infected cells was significantly higher than that in noninfected cells (Fig. 4M). This was in agreement with the results from the proteomics analysis.

**TABLE 3:** Overlapping differentially expressed proteins (COPI-CSFV vs COPI-cell upregulated and COPII-CSFV vs COPII-cell downregulated)

Protein ID	Protein name
A0A287AJZ1	Signal transducer and activator of transcription
A0A286ZPG4	Filamin B
A0A480SBC0	Macrophage-capping protein
A0A0B8RTX3	Adapter protein, phosphotyrosine interaction, and pH domain and leucine zipper containing 1
A0A4X1TNB7	Fascin domain-containing protein
A0A480VDC2	Golgi to ER traffic protein 4 homolog
A0A0B8RSX6	Filamin A, alpha
A0A287AC34	Talin 1
A0A286ZIH3	Elongation factor 1-alpha
A0A4X1SRE7	Clathrin heavy chain
A0A480M011	Fatty acid synthase
A0A287AYY7	Leucine rich repeat-containing 47
A0A4X1VCB2	Eukaryotic translation initiation factor 3 subunit J
A0A0B8S0B1	Adenylyl cyclase-associated protein

**TABLE 4:** Overlapping differentially expressed proteins (COPI-CSFV vs COPI-cell downregulated and COPII-CSFV vs COPII-cell upregulated)

Protein ID	Protein name
I3LRS5	Aldehyde dehydrogenase [NAD(+)]
A0A287BIL8	78 kDa glucose-regulated protein
A0A4X1UF70	GTP-binding nuclear protein Ran
A0A480HAE4	WD repeat-containing protein 1
A0A286ZIN0	Heterogeneous nuclear ribonucleoprotein D
A0A480S836	Glucose-6-phosphate isomerase
A0A4X1UIB6	Hemoglobin subunit alpha
A0A287BMB6	Heterogeneous nuclear ribonucleoprotein A/B
A0A4X1UUB1	Destrin
A0A287ATN8	60 kDa chaperonin
A0A287A3B5	Hydroxysteroid 17-beta dehydrogenase 4
A0A287AGU2	ATP synthase subunit alpha
A0A287B4J2	Adenosylhomocysteinase
A0A287A2C3	Catalase
A0A480X4F2	Vacuolar protein sorting-associated protein 35
A0A286ZND5	Peroxiredoxin-1

### COP I vesicles regulate CSFV RNA replication via mediating FASN transport from the Golgi apparatus to the ER

**Fig 5:**
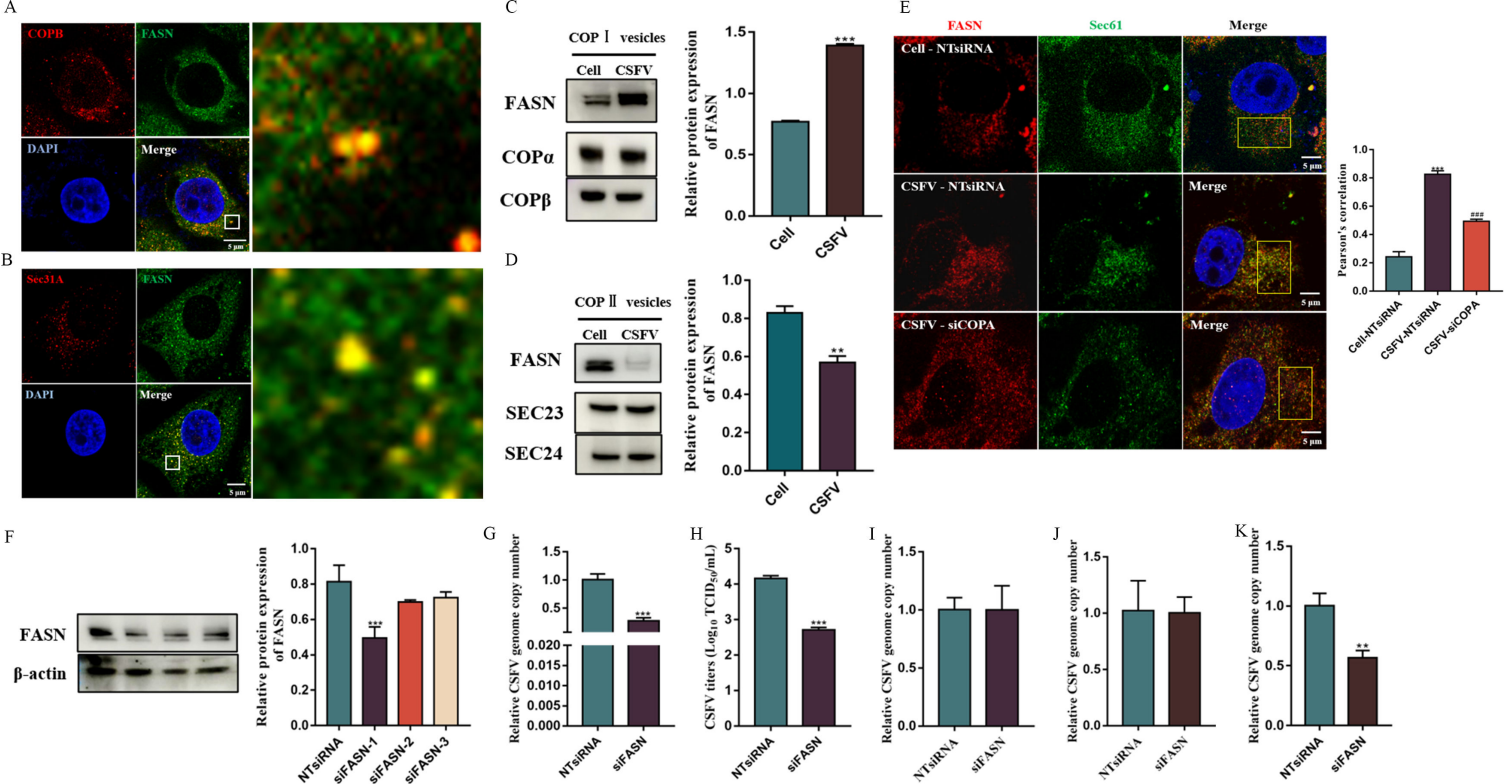
COP I vesicles regulate FASN trafficking from the Golgi apparatus to the ER. (**A**) Colocalization of FASN and COPβ. Cells were infected with CSFV (MOI = 1) for 48 h and stained for FASN, COPβ, and nucleocapsid. Scale bars: 5 µm. (**B**) Colocalization of FASN and Sec31A. Cells were infected with CSFV (MOI = 1) for 48 h and stained for FASN, Sec31A, and nucleocapsid. Scale bars: 5 µm. (**C**) The FASN levels in isolated COP I vesicles from CSFV-infected and uninfected cells were analyzed by Western blot. (**D**) The FASN levels in isolated COP II vesicles from CSFV-infected and uninfected cells were analyzed by Western blot. (**E**) NTsiRNA- and siCOPA-3-transfected cells were infected with or without CSFV (MOI = 1). After 48 h, the cells were stained for FASN, Sec61, and nucleocapsid. Scale bars: 5 µm. (**F**) NTsiRNA-, siFASN-1-, siFASN-2-, and siFASN-3-transfected cells were collected for protein extraction with RIPA lysis, and the FASN expression levels were analyzed by Western blot. (G and H) NTsiRNA- and siFASN-1-transfected cells were infected with 1 MOI CSFV. After 48 h, cells and culture supernatants were collected for CSFV RNA copy numbers and virus titers detection by RT-qPCR and TCID_50_/mL, respectively. (**I**) NTsiRNA- and siFASN-1-transfected cells were infected with CSFV (MOI = 10) in the FBS-free medium for 1 h at 4°C. Unbound virions were then washed away using a pre-cooled citrate buffer solution (pH = 3). Total cells were collected for CSFV RNA copy number detection by RT-qPCR. (**J**) NTsiRNA- and siFASN-1-transfected cells were infected with CSFV (MOI = 10) in the FBS-free medium for 1 h at 4°C to allow virion binding. Cells were then washed with pre-cooled citrate buffer solution (pH = 3) to remove unbound virions and cultured for another 2 h at 37°C. The cells were washed and collected for CSFV RNA copy number detection by RT-qPCR. (**K**) NTsiRNA- and siFASN-1-transfected cells were infected with CSFV (MOI = 1), and CSFV RNA copy numbers were detected by RT-qPCR after 10 h.

FASN is located on the Golgi and participates in viral RNA replication (24–26). Our proteomics analysis showed that FASN was upregulated in COP I vesicles and downregulated in COP II vesicles in CSFV-infected cells. Thus, we hypothesized that CSFV infection induced FASN trafficking from the Golgi apparatus to the ER via COP I vesicles, while inhibiting its release from the ER through COP II vesicles. To test this hypothesis, the colocalization of FASN with COP I and COP II vesicles was determined by confocal microscopy. Images demonstrated that FASN colocalized with COP I and COP II vesicles in CSFV-infected cells (Fig. 5A and B). The relative content of FASN in COP I and COP II vesicles was quantified by Western immunoblotting, and it was revealed that the level of FASN in COP I vesicles from CSFV-infected cells was significantly higher than that from noninfected cells (Fig. 5C), while the level of FASN in COP II vesicles from CSFV-infected cells was significantly lower than that in noninfected cells (Fig. 5D). This was in agreement with the results from the proteomics analysis. Confocal microscopy revealed that CSFV infection recruited FASN to the ER, while inhibition of COP I vesicles impaired FASN transport to the ER (Fig. 5E). These findings clearly demonstrate that CSFV infection enhances FASN transport from the Golgi apparatus to the ER in COP I vesicles and inhibits its movement out of the ER in COP II vesicles.

If COP I vesicles are essential for CSFV RNA replication by recruiting FASN to the ER, and then FASN must play a critical role in CSFV RNA replication. To determine this role, siRNA sequences against FASN were designed and transfected into PK-15 cells. After 48 h, the silencing efficacy was measured by Western immunoblotting assay, and siFASN-1 showed the greatest knockdown efficiency (*P* < 0.001; Fig. 5F). The siFASN-1-transfected cells were inoculated with 1 MOI CSFV for 48 h, and a significant reduction in CSFV proliferation was noted (*P* < 0.001; Fig. 5G and H). To pinpoint the exact role of FASN in CSFV infection, siFASN-1-transfected cells were used to analyze the influence of FASN knockdown on CSFV binding and entry. The results revealed that the number of copies of the CSFV genome was basically unchanged in the siFASN-1-transfected cells, indicating that FASN was not required for CSFV binding and entry (Fig. 5I and J). Next, siFASN-1-transfected PK-15 cells were inoculated with 1 MOI of CSFV for 10 h, after which the cells were collected for CSFV RNA detection. We found that the number of copies of the CSFV genome was lower in the siFASN-1-transfected cells (*P* < 0.01), which meant that FASN participated in CSFV RNA replication (Fig. 5K). Based on these findings, we conclude that COP I vesicles mediated FASN transport from the Golgi apparatus to the ER, thereby supporting CSFV RNA replication.

**Fig 6:**
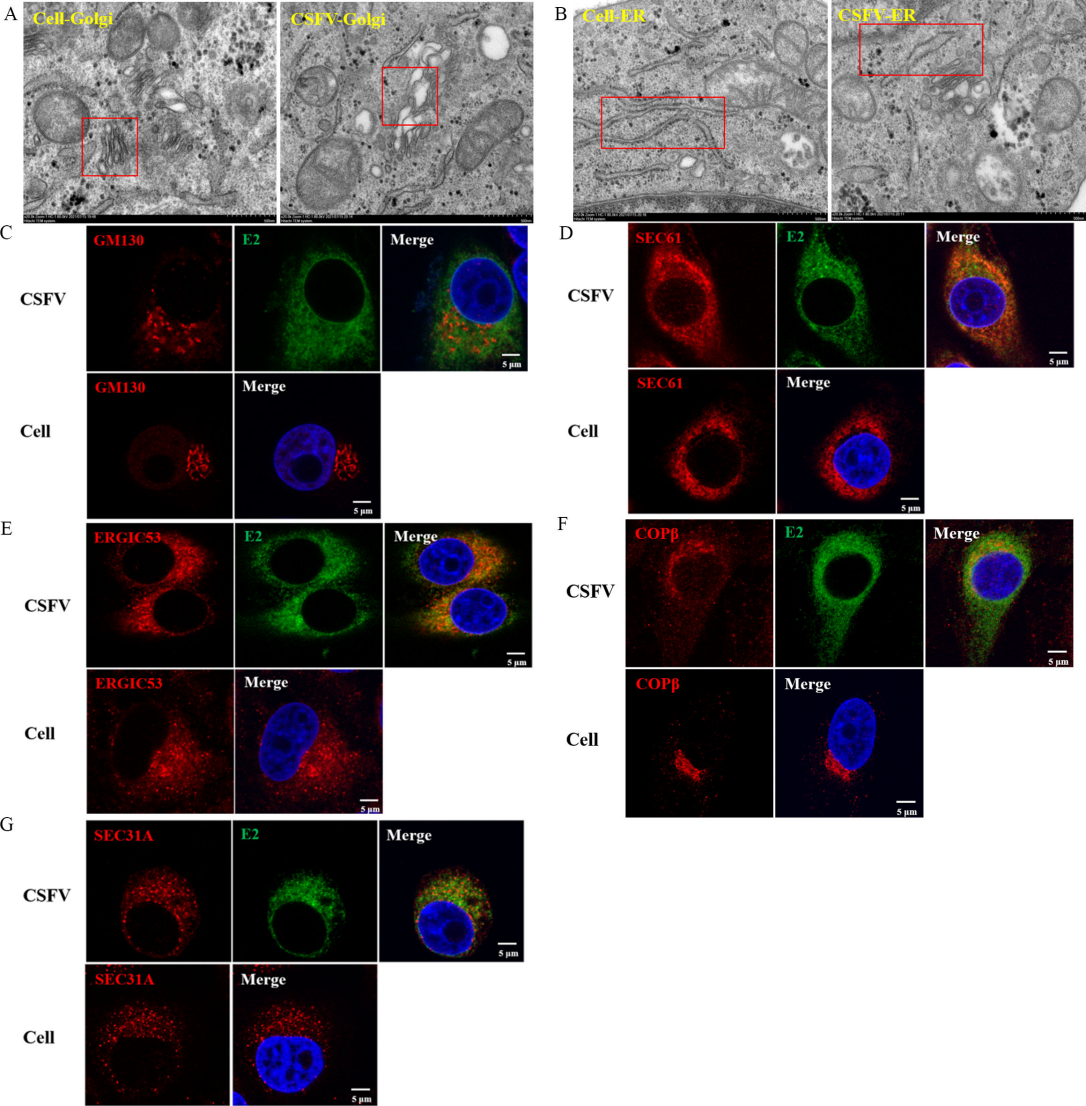
CSFV infection induces morphological alterations of early secretory pathway organelles. (A and B) Electron micrographs of cells infected with or without CSFV (MOI = 10). Boxed areas indicate the Golgi apparatus (1A) or ER (1B), respectively. Scale bars, 500 nm. (**C**) Cells were infected with or without CSFV (10 MOI) for 48 h and stained for a Golgi apparatus marker GM130 and nucleocapsid. Scale bars: 5 µm. (**D**) Cells were infected with or without CSFV (10 MOI) for 48 h and stained for an ER marker Sec61 and nucleocapsid. Scale bars: 5 µm. (**E**) Cells were infected with or without CSFV (10 MOI) for 48 h and stained for the ERGIC marker ERGIC53 and nucleocapsid. Scale bars: 5 µm. (**F**) Cells were infected with or without CSFV (10 MOI) for 48 h and stained for a COP I vesicle marker COPβ and nucleocapsid. Scale bars: 5 µm. (**G**) Cells were infected with or without CSFV (10 MOI) for 48 h and stained for a COP II vesicle marker Sec31A and nucleocapsid. Scale bars: 5 µm.

### CSFV infection remodels the early secretory pathway in PK-15 cells

Since the early secretory pathway is involved in the replication of CSFV, the possibility of an effect of CSFV infection on the early secretory pathway must be considered. Morphological changes in the Golgi apparatus and the ER were observed by TEM after infection with 10 MOI CSFV for 48 h. Compared with the control group, the normal flat stacking structure of the Golgi apparatus in CSFV-infected cells was swollen and fragmented, and the ER structure was also disrupted (Fig. 6A and B). The morphological changes in the Golgi apparatus and ER in CSFV-infected cells were also studied by confocal microscopy, and the results revealed that the Golgi apparatus marker, GM130, was altered from its normal perinuclear aggregation to a scattered distribution in CSFV-infected cells (Fig. 6C). The ER-specific protein, Sec61, was also disrupted from its perinuclear location to a random position in CSFV-infected cells (Fig. 6D). The ERGIC, as mapped by ERGIC53, appeared to be unchanged in CSFV-infected cells (Fig. 6E). The changes indicated that CSFV infection functionally remodeled the structure of the Golgi apparatus and the ER. We also observed that the COPβ protein in COP I vesicles was normally found in the perinuclear region, whereas in CSFV-infected cells, COPβ was dispersed in the cytoplasm (Fig. 6F); in contrast, the Sec31A marker of COP II vesicles from PK15 cells was basically unchanged after CSFV infection (Fig. 6G). These specific morphological changes clearly support our hypothesis that CSFV infection remodels the early secretory pathway.

## DISCUSSION

The early secretory pathway is composed of the ER and Golgi apparatus in concert with a transport system consisting of COP I and II vesicles and is used for cargo trafficking (27). This special intracellular transport system has been exploited by viruses for propagation in host cells (28). There are two main mechanisms in the early secretory pathway that have been shown to be necessary for viral infection. One serves to generate membrane sites for viral ROs and a virion assembly factory and is especially important in the case of SARS-CoV2 (29), dengue virus (DENV) (30), zika virus (ZIKV) (31), HCV (11, 32), and coxsackievirus B3 (CVB3) (33). The other secretory process hijacked by viruses is responsible for the transport of viral proteins and virions and is utilized by SARS-CoV2 (17, 18, 34), HCV (9), human papillomavirus (HPV) (35), Ebola virus (EBOV) (36), and chikungunya virus (CHIKV) (37). In a previous study, we reported that Rab1 and Rab2, which are key regulatory proteins in the early secretory pathway, were involved in the proliferation of CSFV (19, 21). In this study, we demonstrated that disruption of the Golgi apparatus and the ER function using specific chemical inhibitors also impaired CSFV propagation. In addition, we showed that blocking COP I and II vesicle function also inhibited CSFV propagation, supporting the hypothesis that the early secretory pathway is necessary for effective CSFV infection. At the early stage of CSFV infection, both the Golgi apparatus and the ER lost their normal flat vesicle stacking structure, appearing to be swollen and fragmented. We also observed that the COP I transport vesicles were no longer localized to the perinuclear region, but rather were dispersed in the cytoplasm. This remodeling of the early secretory pathway by CSFV infection is consistent with the idea that COP I vesicles play a key role in enhancing CSFV proliferation.

COP I vesicles are composed of α, β, β′, γ, γ′, δ, ε, ζ, and ζ′ subunits, and these organelles are responsible for protein trafficking from the Golgi apparatus to the ER (38). Numerous reports have revealed that COP I vesicles play an essential role in invasion, genome replication, and protein trafficking for many virus families, including *Picornaviridae* (39, 40), *Coronaviridae* (41), and *Flaviviridae* (32, 42). Our previous data supported the hypothesis that COP I vesicles were utilized by CSFV to enter and infect swine umbilical vein endothelial cells (SUVECs) through two different ways (20). Therefore, we examined the role of COP I vesicles in CSFV propagation in PK-15 cells. Different approaches were used, but the conclusions demonstrated the participation of COP I vesicles in CSFV invasion of PK-15 cells and confirmed that CSFV viral RNA replication was inhibited in COP I vesicle inhibitor-treated cells. In other studies, COP I vesicles were found to be required for viral RNA replication of DENV (43), HCV (44), and CHIKV (37). Gazina and colleagues reported that inhibition of COP I vesicles impaired echovirus 11 (EV11) RNA replication and further showed that COP I vesicles appeared to be specifically distributed to the replication complex of EV11, suggesting that the EV11 replication complex appeared to be dependent on the association of COP I vesicles with specific membrane structures (45). Considering that CSFV infection disrupts the distribution of COP I vesicles, we hypothesized that COP I vesicles may serve as a membrane source for the CSFV replication complex. However, confocal microscopy showed that COP I vesicles did not colocalize with CSFV NS5B, a protein involved in viral RNA replication, suggesting that COP I vesicles were not directly involved in the CSFV RNA replication.

Excluding the direct role of COP I vesicles in the CSFV RNA replication, we hypothesized that they could facilitate CSFV RNA replication by transporting protein cargos required for viral RNA replication from the Golgi apparatus to the ER. COP I and COP II vesicles were isolated and analyzed by TEM and Western immunoblotting, and a DIA quantitative proteomics analysis was executed to identify the differences in the proteomes of COP I and COP II vesicles from CSFV-infected cells and noninfected cells. A total of 1,298 proteins were identified and quantified in COP I vesicles and 1,521 proteins in COP II vesicles. Proteins involved in the biogenesis of COP I and II vesicles were identified in the proteome, as well as proteins in the early secretory pathway that constitutively cycle between the Golgi apparatus and the ER. Since CSFV RNA replication occurs in the ER, we hypothesized that during CSFV infection, proteins involved in viral RNA replication may be sequestered in COP I vesicles and transported to the ER, whereas these proteins are reduced or absent in COP II vesicles, resulting in the accumulation in the ER of the factors necessary for CSFV RNA replication. Then, common proteins were upregulated in COP I vesicles by CSFV infection compared to noninfected cells, and the downregulated proteins in COP II vesicles (infected vs noninfected) were analyzed, and fourteen proteins were identified. Among them, FASN, which is located on the Golgi apparatus, is an enzyme that provides energy and as a structural component of cell membrane (46) and is required for viral propagation, particularly in viral RNA replication. In HCV-infected cells, FASN was upregulated and promoted HCV RNA replication through activation of NS5B RNA-dependent RNA polymerase (RdRp) (26). In DENV infection, FASN is relocated to the viral RNA replication site to establish the viral replication complex. Further research showed that Rab18 was required to recruit FASN to the DENV replication complex (25). Heaton and colleagues showed that DENV NS3 recruits FASN to sites of replication and stimulates fatty acid synthesis (47). We and others separately showed that FASN was required for CSFV proliferation in porcine alveolar macrophages and PK-15 cells (24, 48). Liu and colleagues demonstrated that FASN was recruited to CSFV replication sites in the ER and interacted with NS4B to modulate CSFV replication. They also suggested that FASN participated in CSFV replication, possibly through lipid droplet biosynthesis (24). Here, we also found that depletion of FASN inhibited CSFV RNA replication, and blocking COP I vesicle formation decreased the content of FASN on the ER, suggesting COP I vesicles promoted CSFV RNA replication by transporting FASN from the Golgi apparatus to the ER. Although others have argued that COP I vesicles regulated lipid homeostasis through trafficking of adipose triglyceride lipase (ATGL) to the surface of lipid droplets, and through trafficking of sterol regulatory-element binding proteins (SREBPs) (49–51), our study is the first to show COP I vesicle-mediated FASN transport from the Golgi apparatus to the ER. However, further research is needed to elucidate the mechanism of how COP I vesicles regulate FASN trafficking.

In this study, we demonstrated that the cells’ early secretory pathway was required for CSFV proliferation. During CSFV RNA replication, CSFV hijacks COP I vesicles to transport FASN from the Golgi apparatus to the ER, meanwhile inhibiting FASN efflux from the ER via COP II vesicles to promote CSFV RNA replication. However, the specific mechanism by which CSFV manipulates the COP I and II vesicles to regulate FASN trafficking is unknown. In summary, our work not only highlights a new role of COP I vesicles in viral RNA replication but also enriches our understanding of viral exploitation of the early secretory pathway in CSFV proliferation.

## MATERIALS AND METHODS

### Cells and viruses

Porcine kidney-15 (PK-15) cells were obtained from the ATCC (CCL-33) and cultured in Dulbecco’s modified Eagle’s medium (DMEM) (Gibco, 11965092) containing 10% fetal bovine serum (FBS) (Gibco, A5669701) and 1% penicillin-streptomycin (Sigma-Aldrich, TMS-AB2). The cells were grown in an incubator at 37°C with 5% CO_2_. The classical swine fever virus (CSFV; Shimen strain) was obtained from the China Institute of Veterinary Drug Control (Beijing, China).

### Transfection of siRNA

The negative control siRNA (NTsiRNA) and siRNAs targeting COPA, COPD, Sec23, Sec24, and FASN were synthesized by GenePharma (Shanghai, China). Transient transfection of siRNAs was performed using Lipofectamine 2000 (Invitrogen, 11668019), as directed by the manufacturer’s instructions.

### Cell viability assays

Cell viability was measured with a CCK-8 kit (Dojindo, CK04) following the protocol. PK-15 cells were seeded into 96-well plates and incubated with H89 (Beyotime, S1643), Exo1 (TargetMol, T4609), GCA (Selleck, S7266), BFA (Selleck, S7046), tunicamycin (TargetMol, T13229), or control at the indicated concentrations at 37°C in a CO_2_ incubator. After treatment for 48 h, the cell viability reagent (10 µL) was directly added and incubated for another hour at 37°C. The OD_450_ was read using an Infinite M200pro plate reader (Tecan, Männedorf, Switzerland).

### RNA extraction and real-time quantitative PCR (RT-qPCR)

Total RNA was isolated with RNAiso Plus (Takara Bio, Cat 9108) and quantitated with a NanoDrop One (Thermo Fisher Scientific, Waltham, MA, US). RNA was reverse-transcribed into cDNA with the AG EvoM-MLV reverse transcription kit (Accurate Biotechnology, AG11604). CSFV genome copy numbers were normalized to the beta-actin housekeeping gene, using the TB Green Premix Ex Taq II (Takara Bio, Cat RR820A), and tested with the CFX Connect real-time PCR system (Bio-Rad, Hercules, CA, US). Data were analyzed by the 2^-ΔΔct^ method. The primer sequences for RT-qPCR are as follows: CSFV (F: 5′ GAGAAGGACAGCAGAACTAAGC 3′; R: 5′ TTACCGCCCATGCCAATAGG 3′) and β-actin (F: 5′ CAAGGACCTCTACGCCAACAC 3′; R: 5′ TGGAGGCGCGATGATCTT 3′).

### Western blot protein immunoassays

Total protein was isolated from PK-15 cells, COP I, and II vesicles using RIPA lysis buffer containing a 1% protease inhibitor cocktail (MedChem Express). The protein concentration was determined by BCA reaction, and equal amounts were separated by SDS-PAGE on a 4%–20% FuturePAGE gradient gel (ACE Biotechnology, F11420Gel). The proteins were electroblotted to polyvinylidene difluoride (PVDF) membranes (Merck Millipore, ISEQ00010), which were blocked with 5% skim milk, and then incubated with the following primary antibodies: anti-COPα (Abcam, ab192919), anti-ARCN1 (Abcam, ab96725), anti-Sec23 (Abcam, ab179811), anti-Sec24 (Abcam, ab191566), anti-Sec31 (Abcam, ab253009), anti-Sar1 (Abcam, ab125871), anti-FASN (Abcam, ab128870), anti-GRP78 (Proteintech, 11587-1-AP), or anti-β-actin (Abcam, ab8227) at room temperature (RT) for 2 h. After thorough washing, the membranes were incubated with horseradish peroxidase-conjugated goat anti-rabbit IgG (Proteintech, SA00001-2) secondary antibody (1:8000) at RT for 1 h. Lastly, signals were detected by enhanced chemiluminescence and analyzed on a photo documentation system with beta-actin as the internal control.

### Measurement of CSFV titer and replicon number by indirect immunofluorescence analysis (IFA)

CSFV replication (Fig. 1F, 2I and Q) and virus titer (Fig. 1E, 2D, H, L, P, and 5H) were measured by IFA according to a published protocol (20). For CSFV replication, the siRNA-transfected cells or inhibitor-treated cells were fixed in 4% paraformaldehyde (PFA) for 20 min at RT and washed 3 x with PBST. For determining the virus titer, the cells were seeded into 96-well plates and inoculated with cell supernatants containing CSFV for 72 h at 37°C in a 5% CO_2_ incubator. After incubation, the cells were fixed in 4% PFA for 20 min at RT, permeabilized with 0.3% Triton X-100 for 5 min at RT, and washed 3 x with PBST. After blocking with 3% BSA for 2 h, cells were incubated with mouse anti-E2 CSFV antibody (1:200) at RT for 2 h. After three washes with PBST, the cells were incubated with Alexa Fluor 488-conjugated goat anti-mouse IgG H&L antibody (Abcam, ab150113) for 1 h at RT. Fluorescence signals were imaged with a fluorescence microscope (Nikon, Tokyo, Japan), and the virus titer was determined as TCID_50_/mL using the method of Reed and Muench.

### Binding and entry assays

For the binding assay, PK-15 cells transfected with NTsiRNA, siCOPA, siCOPD, or siFASN were infected with 10 MOI CSFV and incubated for 1 h at 4°C to allow the virus to bind but not enter. Unattached virus was removed by washing three times with cold citrate buffer (pH = 3). For the entry assay, the cells were incubated in a fresh culture medium at 37°C for an additional 2 h to allow viral internalization. After the second incubation, the cells were washed with citrate buffer to remove the non-internalized virus, and the cells were collected after washing three times with pre-cooled PBS.

### Confocal microscopy

PK-15 cells were seeded on glass coverslips in 35 mm culture dishes and incubated for 12 h at 37°C. Organelle observation (Fig. 6): cells were infected with CSFV (10 MOI) for 48 h. GM130 and Sec61 distribution (Fig. 1): cells were incubated with 100 nM BFA and 10 µM tunicamycin for 24 h. Colocalization of NS5B with Sec61 and COPβ (Fig. 3): cells were transfected with pEGFP-NS5B for 24 h and then infected with 1 MOI CSFV for 48 h. Colocalization of GRP78 with Sec31A and COPβ (Fig. 4J and K): cells were infected with CSFV (1 MOI) for 48 h. Colocalization of FASN with Sec31A and COPβ (Fig. 5A and B): cells were infected with CSFV (1 MOI) for 48 h . Colocalization of FASN with Sec61 (Fig. 5E): cells were transfected with or without NTsiRNA and siCOPA-3 for 24 h, and cells were infected with 1 MOI CSFV for 48 h.

Cells were washed with cold PBST, fixed in 4% PFA for 20 min, and permeabilized with 0.3% Triton X-100 in PBST for 10 min at RT. After three washes with PBST, the cells were blocked with 3% BSA in PBST for 2 h at RT. The cells were then incubated with the indicated antibodies (GM130, Sec61, COPβ, GRP78, Sec31A, and FASN) for 12 h at 4°C. After three washes with PBS, the cells were incubated with Alexa Fluor 594/488-conjugated goat anti-mouse/rabbit IgG H&L antibody (1:200) for 1 h at RT in the dark. The nuclei were stained with fluorescent DAPI (*blue*) at 37°C for 5 min and washed with cold PBS. Images were obtained by laser-scanning confocal microscopy (LSM510 Meta, Zeiss, Germany).

### Immunoprecipitation of COP I and COP II vesicles

PK-15 cells were inoculated with 10 MOI CSFV for 72 h or sham-infected. The cells were washed with PBS, scraped into cold isotonic extraction buffer, and the cells were disrupted under nondenaturing conditions by forcing the suspension through a syringe with a 21-gauge needle, followed by a syringe with a 27-gauge needle on ice to obtain cell lysates containing intact intracellular vesicles. The cell lysates were subjected to differential centrifugation at 1,000 × *g* for 10 min, 12,000 × *g* for 20 min, and 100,000 × *g* for 1 h at 4°C. The supernatants were collected and concentrated to 2 mL using a centrifugal filter (3 kDa, Millipore) at 4°C. For immunoprecipitation, protein A/G magnetic beads (Selleck, B23201) were pre-incubated with anti-COPα or anti-Sec31A antibody (1:100, Abcam) for 2 h at RT and then incubated with the concentrated supernatant for 12 h at 4°C. Nonspecifically bound antibody was removed by washing three times with an isotonic buffer. Lastly, the COP I and II vesicles were eluted from the magnetic beads by incubation with the COPα or Sec31A peptide (500 µg/mL, Proteintech).

### Electron microscopy

For organelle imaging (Fig. 6A and B), PK-15 cells were inoculated with 10 MOI CSFV for 48 h. The medium was removed, and the cells were detached with a cell scraper and harvested by centrifugation at 1,000 × *g* for 15 min. The cells were fixed with 2.5% glutaraldehyde (Sigma-Aldrich, G6257) at 4°C for 12 h, incubated with 1% osmium tetroxide for 3 h at 4°C, dehydrated in increasing ethanol concentrations (Sigma-Aldrich, E7023) and lastly in acetone (Sigma-Aldrich, 650501), and then embedded in epoxy resin. The samples were serially sectioned with an ultra-thin microtome (Leica) and stained with 2% uranyl acetate (Zhongjingkeyi Technology, GZ02625) and lead citrate (Zhongjingkeyi Technology, GA10701). The stained sections were imaged by TEM at 80 kV (Tecnai G2 Spirit Bio, FEI, US) to observe the Golgi apparatus and endoplasmic reticulum and analyzed for ultra-morphology.

To examine the morphology of COP I and COP II vesicles, 10 µL of isolated vesicles was pipetted onto a carbon-coated copper grid and held for 10 min at RT, after which the excess was removed with filter paper, and the vesicles were shadowed with phosphotungstic acid for 90 s. The samples were examined and imaged with a TEM (Tecnai G2 Spirit Bio, FEI, US) at 80 kV (Fig. 4B).

### Mass spectrometry measurements and analysis

The isolation of COP I and COP II vesicles was carried out essentially as described above. The samples were mixed with SDT buffer (4% SDS, 100 mM DTT, and 150 mM Tris-HCl pH 8.0) and boiled for 15 min. After centrifuging at 14,000 × *g* for 40 min, the protein concentration in the supernatants was measured by BCA protein assay. Equal amounts of protein from all samples were mixed into pooled samples for the establishment of a DDA library using a Q Exactive HF-X mass spectrometer (Thermo Scientific). The peptides in each sample were analyzed by LC-MS/MS operating in the DIA mode by Shanghai Applied Protein Technology Co., Ltd. For DDA library data, the database was searched with Spectronaut software (Spectronaut 14.4.200727.47784). The DIA data were analyzed with Spectronaut software (Spectronaut 14.4.200727.47784) searching the above-constructed spectral library. The differences in group comparisons were assessed according to fold change (FC) in mass-spectrometry data. FC upregulation >1.5 fold or downregulation <0.67 fold and *P* < 0.05 were used as the criteria. The numbers of upregulated and downregulated proteins in each group were compared and tested for significance by *t* test.

### Statistical analysis

All experiments were independently performed at least three times, and data were expressed as mean ± standard deviation (SD). The data were analyzed by *t* test with GraphPad Prism 6 (GraphPad Software, Inc., La Jolla, CA, US). *P* < 0.05 was considered significant. For each experiment, statistical significances are depicted by asterisks in the figures as follows: *P < 0.05, **P < 0.01, and ***P < 0.001.

## Data Availability

The authors confirm that all data supporting the findings of this study are available within the article.

## SUPPLEMENTAL MATERIAL

Tables S1 to S8 jvi.00305-25-s0001.xlsx Identified proteins in COP I and COP II vesicles.

ASM does not own the copyrights to Supplemental Material that may be linked to, or accessed through, an article. The authors have granted ASM a non-exclusive, world-wide license to publish the Supplemental Material files. Please contact the corresponding author directly for reuse.
